# Diagnostic challenge of an arachnoid cyst mimicking hydatid cyst: a case report from Syria

**DOI:** 10.1016/j.ijscr.2025.111373

**Published:** 2025-04-25

**Authors:** Mostafa Jaber Hassan, Ali Ismail, Iyas Salman, Ali Salman, Issam Salman

**Affiliations:** a Department of Neurosurgery, Tartous University, Tartous, Syrian Arab Republic; b Faculty of Medicine, Latakia University, Latakia, Syrian Arab Republic; c Department of Histopathology, Tartous University, Tartous, Syrian Arab Republic

**Keywords:** Arachnoid cyst, Hydatid cyst, Diagnostic challenge, Case report

## Abstract

**Introduction:**

Cystic lesions in the brain, often seen in MRI and CT scans, may arise from various causes, including developmental issues, infections, or tumors. Differentiating between arachnoid cysts and hydatid cysts is critical for effective management, as misdiagnosis can lead to inappropriate treatments and worsen patient outcomes.

**Case presentation:**

An 18-year-old right-handed female from a rural area experienced a right-sided focal seizure lasting two minutes. Her history includes progressive right-hand pain, worsening writing difficulty, persistent headaches, personality changes, and recent memory impairment. She had slurred speech. Radiological examination revealed a massive cystic lesion in the brain which initially appeared to be a hydatid cyst, but histological examination revealed it to be an arachnoid cyst.

**Discussion:**

Arachnoid cysts, comprising about 1 % of intracranial masses, are commonly found fluid-filled sacs located in the arachnoid mater, usually incidentally discovered during imaging. They are more frequent in children and males, often asymptomatic but can cause symptoms based on their location. Classification includes congenital and traumatic types, with congenital being more common. Diagnosis can be challenging, especially when symptoms overlap with conditions like hydatid cysts, complicating treatment decisions.

**Conclusion:**

Arachnoid and hydatid cysts appear similar in surgery and may be hard to distinguish. Dissection difficulty should be the main way to differentiate them. A hydatid cyst diagnosis should not be ruled out until multiple attempts by an experienced surgeon have been made. Further studies are needed to clarify these findings.

## Highlights

• Arachnoid cysts and hydatid cysts may appear completely similar radiographically and macroscopically.
• The difficulty of dissecting the arachnoid membrane is the main factor in changing the diagnosis during surgery.
• The risk of hydatid cyst rupture delays diagnosis changes during surgery, which should be evaluated by an experienced surgeon.

## 1 Introduction

Cystic lesions in the brain are frequently observed in standard imaging tests like magnetic resource imaging (MRI) and computed tomography (CT) scans. These findings can be associated with various causes, including developmental issues, infections, or tumors [1]. These lesions often represent a challenge in diagnosis due to similarity in imaging characteristics (appearance, density, enhancement, and anatomic location) and lack of laboratory tests that confirm or deny the diagnosis [2]. Which sometimes causes a dilemma during the surgery. This has led to a diverse range of clinical presentations caused by elevated intracranial pressure (ICP), Cerebrospinal fluid (CSF) obstruction, and compression of the surrounding neurovascular structures [3]. Cystic lesions of the brain represent a diverse group of conditions that require careful evaluation to determine their nature and appropriate management.

Arachnoid cysts are fluid-filled sacs that form within the arachnoid membrane, one of the three layers of protective tissue surrounding the brain and spinal cord. The management of symptomatic arachnoid cysts may involve surgical intervention, such as cystoperitoneal shunting or fenestration [4,5].

.In contrast, hydatid cysts are a result of infection by the Echinococcus species, a type of parasitic tapeworm. Humans typically become infected through the ingestion of eggs found in contaminated food or water. These cysts can develop in various organs, most commonly the liver and lungs, but can also affect the central nervous system. Diagnosis is primarily through imaging studies, serological tests, and sometimes histopathological examination [6].

.Differentiating between arachnoid cysts and hydatid cysts is crucial for effective management, as their treatment strategies differ significantly. Misdiagnosis can lead to inappropriate treatments that may worsen the patient's condition. This article presents a unique case where an arachnoid cyst was initially misidentified as a hydatid cyst based on imaging characteristics [7]. By examining this case in detail, we aim to highlight the diagnostic challenges faced by clinicians and emphasize the importance of a thorough evaluation when encountering cystic lesions in the central nervous system. This case has been reported in alignment with SCARE checklist 2023 [8].

## 2 Case presentation

An 18-year-old female right-handed patient living in a rural area came to the neurology clinic after a right-sided focal seizure without loss of consciousness involving the right half of the face and extremities lasting for two minutes. In detail in the medical history, the patient mentioned mild progressive pain in the right hand for a year and difficulty in writing that had been progressive for six months and had become more severe a month ago, as she reported dropping things from her right hand. She also suffered from a headache that was not relieved by painkillers and personality changes, as she had become hostile for two months. A slight memory impairment occurred several days ago, which was attributed at the time to psychological stress. The speech was slurred and the patient gave the appearance of being naive. The patient did not report any tobacco or alcohol consumption.

### 2.1 Examination findings

The clinical examination of the cranial nerves revealed normal function except for the seventh nerve, which showed weakness in the expressive muscles of the lower half of the face, indicating partial central facial paralysis.

In the upper right limb assessment, muscle tone and reflexes were normal; however, muscle strength showed weakness with the wrist flexors rated at 4- and the biceps brachii at 4+ according to Manual Muscle Testing (MMT). The upper left extremity tests were normal. A tactile stereognosis test yielded positive results on the left side and negative on the right side. For the lower limbs, both reflexes and muscle tone were normal, but there was mild muscle weakness present in the left extremity.

The visual examination indicated papilledema on the left side. Overall, the examination of the remaining systems was within normal limits.

### 2.2 Radiological imaging

**Fig. 1:**
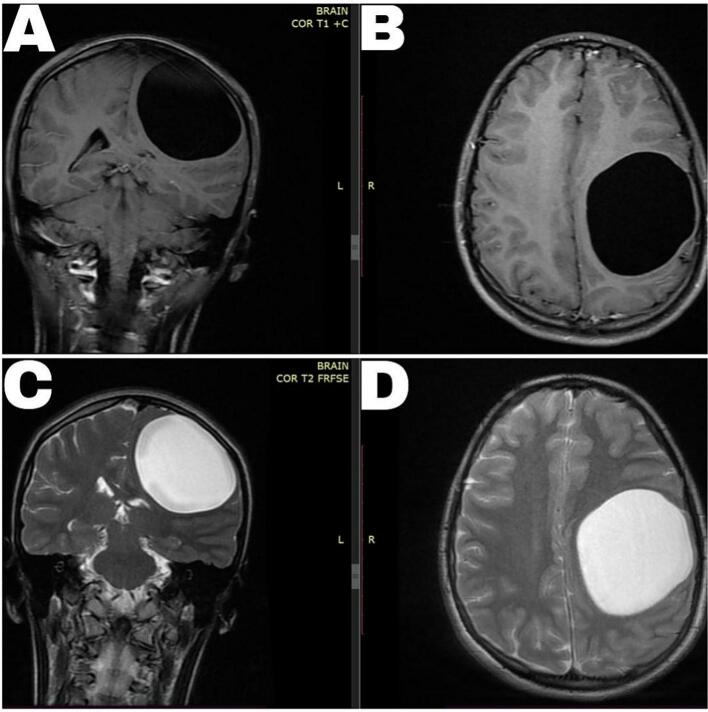
Pre-operative brain MRI with contrast. (A) T1 Coronal, (B) T1 Axial, (C) T2 Coronal and (D) T2 Axial. Reveal a well-defined, thin-walled cystic mass located in the left frontal, temporal, and parietal lobes. The mass is hypointense compared to the cerebral cortex on T1-weighted sequences and hyperintense on T2-weighted sequences.

A brain MRI with contrast revealed a well-defined, thin-walled cystic mass located in the left frontal, temporal, and parietal lobes, measuring approximately (6.79, 6.80, 6.10) cm. The mass was hypointense compared to the cerebral cortex on T1-weighted sequences and hyperintense on T2-weighted sequences. There was a noticeable midline shift to the right, but no hydrocephalus was observed, and all other brain structures appeared normal. An anteroposterior chest X-ray and a liver ultrasound both returned normal results. However, the shape and texture of the lesion identified in the MRI suggested a preliminary diagnosis of a hydatid cyst (Fig. 1).

### 2.3 Surgical procedure

Under general Anesthesia, the patient was positioned supine with her head secured in a Mayfield clamp. A wide craniotomy flap was created on the left frontal, temporal, and parietal bone. The dura mater was opened in a U-shape, exposing the arachnoid membrane covering the cyst. In accordance with the protocol for handling a hydatid cyst, we attempted to dissect the arachnoid membrane from the borders of the cyst adjacent to the frontal lobe. The dissection was challenging; however, we did not rule out the diagnosis of a hydatid cyst due to the risk of rupture and the absence of visual indications suggesting an alternative diagnosis. After 40 min of maneuvering and gradually increasing the severity of the maneuvers, the cyst ruptured, and its contents were aspirated with a pipette. Notably, no daughter cysts were observed. An incision was then made in the center of the cyst to facilitate its removal. However, there was no visible parietal complex, which raised suspicion for an alternative diagnosis: an arachnoid cyst. Based on this new diagnosis, the surgical procedure continued with the removal of the arachnoid membrane's adhesions to the brain matter. Subsequently, the arachnoid membrane itself was excised. The dura was then closed using vicryl sutures, and the bone flap was reattached and secured.

A bipolar coagulator was utilized to control any bleeding.

### 2.4 Histopathological examination

**Fig. 2:**
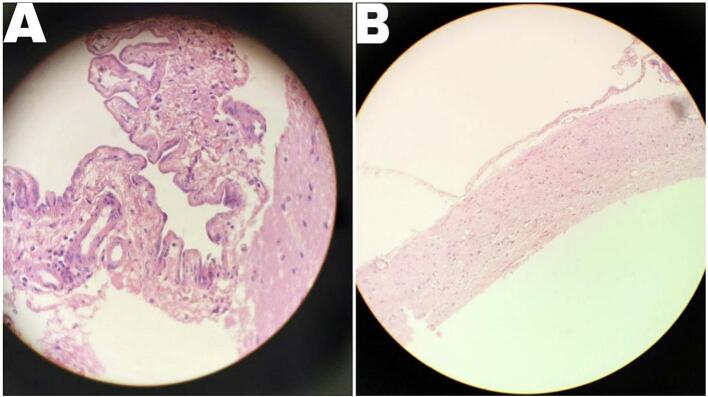
Histopathological examination. (A) The cyst wall shows connective tissue lined by one row of cubic meningothelial cells, which is characteristic of an Arachnoid cyst. (B) Showing some glial cells due to the resection site in the brain.

Histopathological examination of the cyst wall showed connective tissue lined by one row of cubic meningothelial cells, which was characteristic of an Arachnoid cyst (Fig. 2).

### 2.5 Outcomes and Follow-up

The initial evaluation after the surgery indicated a deterioration in the patient's condition, particularly in muscle strength in the right upper limb 8/10. Additionally, memory was noticeably affected, stuttering increased slightly, and the patient developed dysgraphia. However, the pain in the right hand and headaches improved immediately following the surgery.

**Fig. 3:**
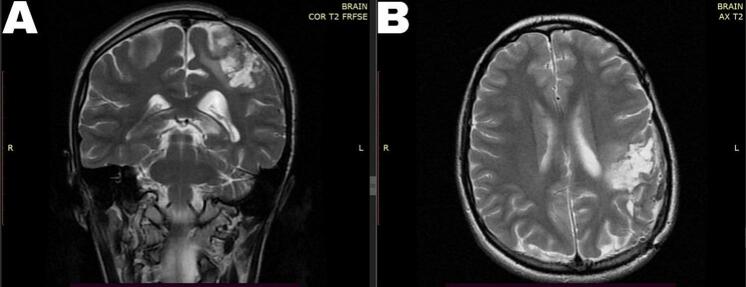
Post-operative brain MRI after six months. (A) T2 Coronal, (B) T2 Axial. Showing good redistribution of the brain without any signs of recurrence.

Within six months, the patient demonstrated a significant gradual improvement in all her symptoms. Muscle strength increased to a score of 4+, memory returned to normal, dysgraphia resolved, and MRI results showed good redistribution of the brain without any signs of recurrence (Fig. 3).

A follow-up conducted one year after the surgery revealed almost complete improvement. There were no signs of memory or speech disorders, and muscle strength had greatly improved. The only residual issue noted during the patient’s examination was tactile agnosia.

## 3 Discussion

Arachnoid cysts, representing approximately 1 % of all intracranial masses, are the most frequently encountered cystic lesions within the cranium, distinguished by their delineated, extra-axial location which look like fluid-filled sacs that resemble cerebrospinal fluid (CSF) and form within the arachnoid mater, one of the membranes surrounding the brain and spinal cord, frequently discovered incidentally during neuroimaging studies, such as CT or MRI, as the majority of these lesions remain asymptomatic [4,7]. While often diagnosed in pediatric patients, who are also more prone to symptomatic presentation, adult symptomaticity is relatively infrequent, occurring in approximately 5.3 % of cases. Additionally, males are approximately twice as likely to develop these cysts compared to females. There is no evidence suggesting that the risk of developing arachnoid cysts increases with age [5,9].

.The likelihood of symptom development varies based on cyst location. Middle cranial fossa and retrocerebellar cysts are less likely to produce symptoms compared to suprasellar and quadrigeminal cysts, which have a greater propensity for clinical manifestations. This was demonstrated in our case, as the difficulty in using the right hand and the tendency to drop objects can be attributed to the pressure exerted by the lesion on the area responsible for voluntary movement in the right side of the body, which corresponds to area 4 according to Brodmann's classification [10]. As for the personality, behavioral, and speech disturbances, they were attributed to their effect on the frontal lobe and the areas it contains [11]. Additionally, the headache and papilledema observed in the patient resulted from the mass effect described previously. On the other hand, some symptoms, such as seizures, are uncommon in association with arachnoid cysts [12], but yet they were one of the late symptoms that occurred in our patient.

Arachnoid cysts can be generally classified into two categories: congenital (primary) and traumatic (secondary). However, this classification does not cover all possible causes. Primary arachnoid cysts are the more common type and develop due to congenital factors. In contrast, secondary cysts can result from trauma, as well as from iatrogenic causes, infections, and other conditions like intracranial hemorrhage [13,14]. Given the patient's young age and the absence of any history of head trauma, infection, or bleeding, we suspect a congenital cause. The detailed radiograph also revealed slight asymmetry in the skull's axial view, which suggests a congenital mechanism such as arachnoid duplication.

In this case, the patient’s rural background raised initial suspicion for a hydatid cyst, a consideration that is particularly relevant given the epidemiological association of hydatid disease with certain geographic areas [6]. Imaging studies revealed a lesion that presented with characteristics more typical of a hydatid cyst, displaying regular, almost circular edges without any relationship to the fissure of Sylvian, which is often seen in arachnoid cysts located in the middle fossa [6,15,16]. However, it is crucial to recognize that there are no definitive imaging features on MRI or CT that can reliably differentiate between an arachnoid cyst and a hydatid cyst, contributing to diagnostic ambiguity [16,17]. Although laboratory tests did not confirm the presence of a hydatid cyst, such results do not entirely exclude the diagnosis [18,19]. Furthermore, cerebral convexity arachnoid cysts (CCACs), which occur over the cerebral hemispheres, constitute a relatively small subset of clinically observed arachnoid cysts, further complicating the differential diagnosis in this scenario [20]. Therefore, considering these factors, we conclude that the most likely diagnosis, in this case, is a hydatid cyst, despite the overlapping imaging characteristics with arachnoid cysts.

The continued adoption of the diagnosis of a hydatid cyst even after the dura mater is opened is due to the lack of any visual differences in the surgical field between it and an arachnoid cyst. The surface layer of both is the arachnoid membrane. Furthermore, the presence of daughter cysts attached to the surface cannot be relied upon, as they are not present in all cases. The difficulty of dissection is the primary key to differentiating between these two cysts. The arachnoid membrane is relatively easy to dissect in the case of a hydatid cyst, compared to the inability to dissect at all in the case of an arachnoid cyst. However, the risk of cyst rupture and the spread of daughter cysts prevents even experienced surgeons from changing the diagnosis and adopting the alternative diagnosis of an arachnoid cyst.

As we found during the follow-up after the surgery, the increase in muscle weakness, memory disturbance, and dysgraphia were attributed to the traction on the adjacent structures during the attempt to separate the edges of the cyst from the brain tissue. These symptoms subsequently decreased over time because there was no apparent damage to the brain and were limited to traction alone.

## 4 Conclusion

The appearance of an arachnoid cyst and a hydatid cyst is very similar in the operating field and may not be distinguishable even radiographically. Therefore, the difficulty of dissection should be considered the primary criterion for differentiating between them when other criteria are equal. The diagnosis of a hydatid cyst should not be overlooked until multiple dissection attempts by an experienced surgeon have been made. Further studies are needed to clarify these findings.

## CRediT authorship contribution statement

**Mostafa Jaber Hassan:** is the first author, contributed to drafting, editing, reviewing, data collection, and analysis, and assisted in the surgical operation as a co-surgeon. The author reviewed and accepted the paper.

**Ali Ismail:** contributed to drafting, editing, reviewing, data collection, and analysis. The author reviewed and accepted the paper.

**Iyas Salman:** contributed to drafting, editing & reviewing. The author reviewed and accepted the paper.

**Ali Salman:** contributed to confirming the histopathological examination.

**Issam Salman:** Is the supervisor**,** contributed to reviewing, and assisted in the surgical operation as a Surgeon. The author reviewed and accepted the paper.

## Consent

Written informed consent was obtained from the patient for publication and any accompanying images. A copy of the written consent is available for review by the Editor-in-Chief of this journal on request.

## Ethical approval

Our institutions do not require ethical approval for reporting individual cases or case series.

## Guarantor

**Mostafa Jaber Hassan** accepted full responsibility for the work, had access to the data, and controlled the decision to publish.

## Sources of funding

The authors received no financial support for the research, authorship, and/or publication of this article.

## Declaration of competing interest

All authors declare that there are no conflicts of interest.

## References

[1] Taillibert S., Le Rhun E., Chamberlain M.C. (2014). Intracranial cystic lesions: a review. Curr. Neurol. Neurosci. Rep..

[2] Oprişan A., Popescu B.O. (2013). Intracranial cysts: an imagery diagnostic challenge. ScientificWorldJournal.

[3] Bothwell S.W., Janigro D., Patabendige A. (2019). Cerebrospinal fluid dynamics and intracranial pressure elevation in neurological diseases. Fluids Barriers CNS..

[4] Pradilla G., Jallo G. (2007). Arachnoid cysts: case series and review of the literature. Neurosurg. Focus.

[5] Al-Holou W.N., Terman S., Kilburg C., Garton H.J., Muraszko K.M., Maher C.O. (2013). Prevalence and natural history of arachnoid cysts in adults. J. Neurosurg..

[6] Wen H., Vuitton L., Tuxun T. (2019). Echinococcosis: advances in the 21st century. Clin. Microbiol. Rev..

[7] Pereira R.G., Ribeiro B.N.F., Hollanda R.T.L., de Almeida L.B., Simeão T.B., Marchiori E. (2021). Non-neoplastic intracranial cystic lesions: not everything is an arachnoid cyst. Radiol. Bras..

[8] Sohrabi C., Mathew G., Maria N. (2023). The SCARE 2023 guideline: updating consensus surgical CAse REport (SCARE) guidelines. Int. J. Surg..

[9] White ML, Das JM. Arachnoid cysts. In: *StatPearls*. Treasure Island (FL): StatPearls Publishing; February 2, 2024.

[10] Damiani D., Nascimento A.M., Pereira L.K. (2020). Cortical brain functions – the Brodmann legacy in the 21st century. Arq Bras Neurocirurgia..

[11] Shettar M., Karkal R., Misra R., Kakunje A., Mohan Chandran V.V., Mendonsa R.D. (2018). Arachnoid cyst causing depression and neuropsychiatric symptoms: a case report. East. Asian Arch. Psychiatry.

[12] de Longpre J. (2017). Large arachnoid cyst. N. Engl. J. Med..

[13] Mustansir F., Bashir S., Darbar A. (2018). Management of arachnoid cysts: a comprehensive review. Cureus.

[14] STARKMAN SP, BROWN TC, LINELL EA. Cerebral arachnoid cysts. J. Neuropathol. Exp. Neurol. 1958;17(3):484-500. doi:10.1097/00005072-195807000-00009. 13564260

[15] Erman T., Gocer A.İ., Tuna M., Ergin M., Zorludemir S., Cetinalp E. (2004). Intracranial arachnoid cysts: clinical features and management of 35 cases and review of the literature. Neurosurg. Q..

[16] Galassi E., Tognetti F., Gaist G., Fagioli L., Frank F., Frank G. (1982). CT scan and metrizamide CT cisternography in arachnoid cysts of the middle cranial fossa: classification and pathophysiological aspects. Surg. Neurol..

[17] Okur A., Ogul H., Sengul G., Karaca L., Nalbantoglu N.G., Kantarci M. (2014). Magnetic resonance spectroscopy and magnetic resonance imaging findings of the intracerebral alveolar echinococcosis. J. Craniofac. Surg..

[18] Tuzun Y., Kadioglu H.H., Izci Y., Suma S., Keles M., Aydin I.H. (2004). The clinical, radiological and surgical aspects of cerebral hydatid cysts in children. Pediatr. Neurosurg..

[19] Debourgogne A., Goehringer F., Umhang G. (2014). Primary cerebral alveolar echinococcosis: mycology to the rescue. J. Clin. Microbiol..

[20] Srinivasan W., Maurer A., Thorell W., Snow E.L. (2024). Cerebral convexity arachnoid cysts: a focused systematic review with defining characteristics. Transl Res Anat..

